# Chagas Disease: A Silent Threat for Dogs and Humans

**DOI:** 10.3390/ijms25073840

**Published:** 2024-03-29

**Authors:** João Durães-Oliveira, Joana Palma-Marques, Cláudia Moreno, Armanda Rodrigues, Marta Monteiro, Graça Alexandre-Pires, Isabel Pereira da Fonseca, Gabriela Santos-Gomes

**Affiliations:** 1 Global Health and Tropical Medicine, GHTM, Associate Laboratory in Translation and Innovation Towards Global Health, LA-REAL, Instituto de Higiene e Medicina Tropical, IHMT, Universidade NOVA de Lisboa, UNL, Rua da Junqueira 100, 1349-008 Lisbon, Portugal; joaodo@ihmt.unl.pt (J.D.-O.); santosgomes@ihmt.unl.pt (G.S.-G.); 2 Centre for Interdisciplinary Research in Animal Health, CIISA, Faculty of Veterinary Medicine, FMV, University of Lisbon, ULisboa, 1649-004 Lisbon, Portugal; gpires@fmv.ulisboa.pt (G.A.-P.); ifonseca@fmv.ulisboa.pt (I.P.d.F.); 3 Associate Laboratory for Animal and Veterinary Sciences (AL4AnimalS), 1300-477 Lisbon, Portugal

**Keywords:** *Trypanosoma cruzi*, Chagas disease, zoonotic infection, reservoir, dogs, medical advances, clinical signs, vaccines

## Abstract

Chagas disease (CD) is a vector-borne Neglected Zoonotic Disease (NZD) caused by a flagellate protozoan, *Trypanosoma cruzi*, that affects various mammalian species across America, including humans and domestic animals. However, due to an increase in population movements and new routes of transmission, *T. cruzi* infection is presently considered a worldwide health concern, no longer restricted to endemic countries. Dogs play a major role in the domestic cycle by acting very efficiently as reservoirs and allowing the perpetuation of parasite transmission in endemic areas. Despite the significant progress made in recent years, still there is no vaccine against human and animal disease, there are few drugs available for the treatment of human CD, and there is no standard protocol for the treatment of canine CD. In this review, we highlight human and canine Chagas Disease in its different dimensions and interconnections. Dogs, which are considered to be the most important peridomestic reservoir and sentinel for the transmission of *T. cruzi* infection in a community, develop CD that is clinically similar to human CD. Therefore, an integrative approach, based on the One Health concept, bringing together the advances in genomics, immunology, and epidemiology can lead to the effective development of vaccines, new treatments, and innovative control strategies to tackle CD.

## 1. Introduction

Chagas disease (CD) or American trypanosomiasis, caused by a flagellated protozoan, *Trypanosoma cruzi*, is one of the most Neglected Zoonotic Diseases (NZD). According to recent data, CD has an annual incidence of 30,000 new cases in 21 Latin American countries, affecting nearly 6 million people, and causing on average 12,000 deaths annually. Furthermore, an estimated 8600 newborns become infected during gestation [1]. The efforts of the last decades have resulted in vectorial control in Central America. However, while the prevalence has been reduced in endemic areas, a significant increase in non-endemic countries has been observed due to the massive influx of Latin American migrants to Asia, North America, Oceania, and Europe, especially to Spain, Portugal, and Italy [2,3], making the disease a global health issue. In non-endemic countries, blood transfusion, organ transplantation, or vertical transmission from mother to child are the main forms of transmission of infection [4]. Addressing CD is also challenging due to the heterogeneity of healthcare systems and a substantial number of underdiagnosed and undertreated individuals in non-endemic areas [3]. The diagnosis depends on the disease’s stage and, classically, two clinical phases are defined—acute and chronic [5]. The first is difficult to characterize clinically as it is usually asymptomatic and, if there are manifestations, they are transient and non-specific, so tend to be overlooked/disregarded [6]. The chronic phase is usually associated with permanent alterations in the nervous and digestive systems as well as severe cardiac modifications [6,7] and may last the patient’s entire life [8].

*T. cruzi* infections affect a diverse variety of hosts including humans and domestic animals, such as horses, pigs, cats, and dogs. Moreover, the multifaceted nature of human–animal relationships is constantly evolving, influenced by climate changes, anthropogenic impacts and natural factors. The increase in travel and tourism and the international trade of live animals as pets or as part of breeding programs for endangered wildlife species constitute major factors impacting the epidemiology of CD and posing important challenges to veterinary sciences [9]. The peridomestic cycle of the parasite plays an important role since infection of those species can indicate the presence of an active *T. cruzi* transmission cycle and represent an increased risk for human infection, as observed in Latin America and the United States of America [10,11]. Also, in recent years, *T. cruzi*, traditionally considered a vector-borne disease (VBD), has been found to be able to infect humans through the ingestion of contaminated food/drink, elevating CD to a global challenge due to large-scale food production, processing, and worldwide distribution [12]. Research on *T. cruzi* has been focused on developing new treatment and prophylaxis strategies, understanding the biology and genetics of the parasite, and investigating the transmission dynamics of CD. Still, there are few treatment options and no commercially available diagnostic tests to detect *T. cruzi* infections in dogs [13]. Besides that, at present time, there is no prophylactic or therapeutical vaccine against human or canine CD.

## 2. The Dog’s Role in *T. cruzi* Complex Life

**Figure 1:**
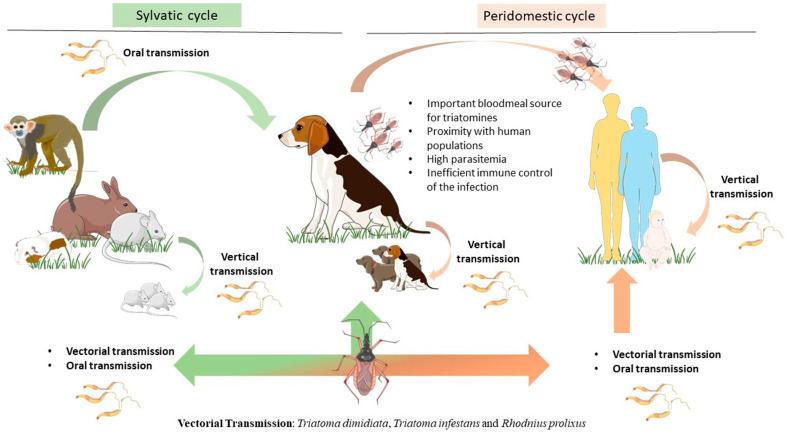
Epidemiological importance of the dog as the main peridomestic reservoir of *T. cruzi* parasites. Dogs can be infected by *T. cruzi* directly by vectorial transmission. However, triatomines can feed on a huge variety of mammals, including humans and dogs as well as sylvatic animals such as rats, guinea pigs, monkeys, raccoons, and armadillos. Infected mammals can transmit *T. cruzi* by vertical transmission, perpetuating the parasite cycle. In more recent years, a new transmission route by ingestion of infected animal tissue or vector-contaminated food or drinks has emerged as a major transmission route for *T. cruzi*. Dogs play a key role as peridomestic reservoirs of *T. cruzi* parasites, as they live close to humans, constitute an important bloodmeal source for triatomines, and also are susceptible hosts to *T. cruzi* infection. The figure was partly drawn using Servier Medical Art, provided by Servier, licensed under a Creative Commons Attribution 4.0 unported license (https://creativecommons.org/licenses/by/4.0/).

The protozoan *T. cruzi*, the causative agent of CD, has a complex life cycle not only due to the parasite’s ability to infect a wide variety of mammals but also to the multiple ways of transmission (Figure 1). Extensive environmental changes, such as urbanization and deforestation in CD endemic areas, increased the likelihood of outbreaks [14]. The most studied form of CD transmission is vectorial, by the triatomine, also known as the ‘kissing bug’. The triatomine can effectively infect more than 180 species of mammals [2,15], *Triatoma dimidiata*, *Triatoma infestans* and *Rhodnius prolixus* being the most medically concerning species in Central and South America [16,17,18,19]. These hematophagous and nocturnal insects feed on humans, domestic animals such as dogs and cats, or wild animals, including armadillos, raccoons, and rats [20,21,22].

The vectorial transmission is initiated when triatomine (either male or female) feeds on an infected host and ingests blood trypomastigotes (BTs). In the insect’s midgut, BTs differentiate into highly replicative epimastigote forms. The differentiation from epimastigotes into infective forms (metacyclic trypomastigotes, MTs, metacyclogenesis) is accomplished only in the rectal ampulla, from where MTs are excreted within the feces. When this infected triatomine feeds again from another mammal, it deposits its MT-contaminated feces near the feed-borne wound [23,24]. By defecating while feeding, the triatomine creates in the host an entry point for the infectious parasite, potentiating the chance of infection (stercorarian transmission) [25]. Once in the host, MTs enter the mammal’s blood, invade host cells (macrophages, smooth and striated muscle cells), and begin their intracellular progression from MTs into amastigotes (AMs) [26,27]. AMs multiply by binary fission in the cell’s cytoplasm and differentiate into BTs. Such multiplication causes cell rupture, freeing these forms into the mammal’s bloodstream and allowing them to infect new cells [28]. AMs can establish themselves in muscle tissue, causing several clinical signs associated with the chronic phase of the disease [23].

When it comes to dogs, it is not clear that vectorial transmission is the most common route on the peridomestic cycle. Many authors suspect that oral transmission, either by directly eating the infected vectors, ingesting meat from infected mammals, or feeding on a parasite-infected lactating mother, might be the most frequent [29,30]. The oral-transmitted cycle begins with the intake of elements containing either macerated triatomines or contaminated secretions [31]. Several regions have reported cases of *T. cruzi* infection outbreaks in human populations linked to the ingestion of drinks and food contaminated with infected mammals’ secretions or triatomine feces [32,33,34]. Once in the mammal’s mouth or nose cavity, the parasites’ transalidases can adhere to the sialic acids in the palate or go through the digestive tract. If they stick to the palate, they efficiently replicate in the nasal cavity, enter the bloodstream or nasal nerves, and have the direct possibility to invade and establish into the muscle tissue (cardiomyocytes, for example) as AMs. If they reach the stomach, MTs adhere to the gastric mucosal epithelium and make their way to other organs. *T. cruzi* can now invade surrounding cells, transform into AMs, multiply, and generate permanent pseudocysts or differentiate again and infect new cells [33,35,36]. Both pathways most certainly lead to chronic CD once the AMs form enough pseudocysts, causing many life-threatening manifestations such as organomegaly and cardiac dysfunctions, depending on which tissues are parasitized [29,37,38].

Within the realm of animal sentinel systems, particularly dogs, there is a prominent role in using them for assessing human exposure and environmental risks and monitoring complex ecosystems. The suitability of an animal species as a sentinel hinges on various critical characteristics. Ideally, an effective sentinel should exhibit susceptibility to the pathogen and its responses should be quantifiable. Additionally, it should occupy a territorial domain or “home range” that aligns with the target monitoring area. The sentinel species should be readily accessible, easily countable, and capture-friendly. Moreover, a significant population size or density is essential to enable the collection of representative samples. These criteria underscore the importance of selecting the most appropriate sentinel species for successful environmental surveillance and risk assessment endeavors, ultimately improving both human and dog living conditions [39]. In this context, dogs are considered simultaneously the most important peridomestic reservoir and sentinel for the transmission of *T. cruzi* infection. They live in close proximity to humans, constituting an important bloodmeal source for triatomine bugs, and they are also highly susceptible to *T. cruzi* infection [40]. Additionally, exhibits high parasitemia in the acute phase of the disease [41], mostly associated with a deficient innate immune response. Due to prolonged outdoor staying, where vectors could be present, shelter and stray dogs are more likely to encounter *T. cruzi*. Moreover, wildlife species involved in *T. cruzi* transmission often inhabit areas around human dwellings, where dogs may come into contact with infected vectors or even preying on infected mammals [42,43]. Interestingly, *T. cruzi* infections do not show strong breed associations, indicating that any exposed dog is susceptible to infection [44]. Thus, the reservoir competence of dogs for *T. cruzi* seems to be related not only to their living conditions but also to age and poor external clinical aspect (ECA) based on nutritional condition parameters, such as the degree of muscle development, external evidence of bone structures, state of the animal hair, and existence of fatty deposits. According to Petersen and colleagues [45], dogs with a bad ECA had a 2.6 to 6.3 times greater probability of infecting *T. infestans* after a bloodmeal, which strongly contributes to increasing the transmission risk inside human dwellings [46,47,48].

## 3. *T. cruzi:* A Parasite with Multiple Identities?

Considered by many to be a very taxonomically diverse species, incorporating a vast group of strains, the CD causative agent has been classified as a protozoan belonging to the Kinetoplastea class, Trypanosomatida order, Tripanosomatidae family, *Trypanosoma* genus and *Trypanosoma cruzi* species [49]. Antigenic variation is one of the abilities demonstrated by this parasite to evade the mammal’s host immune system. This mechanism occurs through the alteration of its surface glycoproteins, using plenty of molecules such as mucins, gp63 peptidases, mucin-associated surface proteins (MASPs), dispersed gene family 1 (DGF-1), and trans-sialidases enzymes (TS). TS is considered the most important molecule in this process. More specifically, by acquiring sialic residues from host glycoconjugates and installing them in the form of a coat of sialylated molecules, the parasite mimics the host’s glycocalyx. This maneuver constitutes a crucial parasite survival strategy and demonstrates its close adaptation to the mammal host. The genetic diversity of *T. cruzi* is compiled into seven discrete typing units (DTUs), from TcI to TcVI and TcBat, each one of these being defined by Veláquez-Ortiz and Ramírez (2020) [33] as “a group of strains that share genetic features and can be identified using a set of specific genetic markers”.

The regulation of TS enzymes’ expression has been proven to be different across the known DTUs. This regulation occurs both by limiting the amount of mRNA that is being produced and by post-transcriptional processes. Some DTUs present elevated TS expression, for example, TcI and TcII. Meanwhile, TcIII and TcIV appear to have lower expression of TS [50,51]. Quantitative differences in TS expression patterns across the seven DTUs appear to be at the center of antigenic variation and CD clinical manifestations [50] Thus, it is proposed by some authors that the differences in clinical manifestations caused by each DTU are probably due to the variation in TS expression [52,53,54].

Currently, in endemic countries, the genotypes TcI, TcII, TcV, and TcVI are the most commonly found causing American human trypanosomiasis [55,56]. Meanwhile, when it comes to canine trypanosomiasis, the genotypes TcI and TcIV, shared with humans are the ones found to be the cause of the disease [48,57]. As demonstrated in Table 1, all *T. cruzi* DTUs identified in the sylvatic reservoirs are already found in the canine and in the human population. As such, *T. cruzi* identification in dogs is considered as an indicator of the presence of Chagas disease across diverse biotopes and regions. However, the true potential of dogs as sentinels for *T. cruzi*, could be more explored, which is essential for mapping *T.cruzi* DTUss natural distribution across different regions and anticipating the introduction of new DTU in the community.

As discussed before, CD presents complex epidemiological variables regarding the transmission and a few examples of field studies performed throughout the Americas illustrate the epidemiological situation regarding canine CD. Several studies in the Southwestern United States demonstrated that *T. cruzi* actively circulates through vector, wildlife, and domestic dog populations [65,66,67]. Research conducted in Texas, Oklahoma, and Louisiana has revealed prevalence rates of dog infection ranging from 3.6% to 22.1%, and reaching as high as 71.0% in certain multi-dog kennels, underscoring the significant burden of Chagas disease among domestic dogs [42,65,66,67,68,69,70]. Moreover, in Texas, 54.4% of triatomines were infected with *T. cruzi,* the prevalence of infection being higher in adults and males (58.7%) than in nymphs (11.3%). Additionally, triatomines were infected with discrete typing units TcI and/or TcIV [71].

However, countries such as Argentina, Colombia, Panamá, and Venezuela have predominantly an enzootic cycle, in which dogs are pivotal for the transmission dynamics and the emergence of new epidemiological scenarios [46,48,59,62]. In Brazil, the epidemiological relevance of the dog population needs to be deepened, since, according to the state, the *T. cruzi* seroprevalence ranges from not reported to 53% as recently reviewed by Freitas and colleagues (2022) [13]. Thus, the development of diagnostic tools to identify *T. cruzi* infection in dogs and other domestic animals is urgently needed. It is also worth noting that the migration of dogs from endemic to non-endemic regions may introduce a veterinary health challenge, as infected dogs may exhibit clinical symptoms in areas where veterinarians are less experienced in recognizing signs of Chagas disease.

## 4. Immunopathogenesis and Clinical Manifestations Chagas Disease

**Figure 2:**
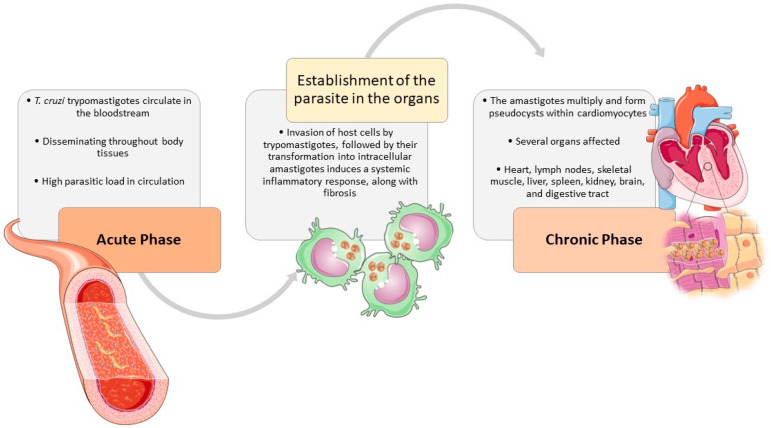
The natural course of *T. cruzi* infection in the host. The clinical signs, associated with *T. cruzi* infection, are shared by different affected species, especially humans and dogs. During the acute phase, *T. cruzi* trypomastigotes circulate in the bloodstream and can enter macrophages, disseminating throughout most body tissues The invasion of host cells, triggers trypomastigotes transformation into intracellular amastigotes and induces a systemic inflammatory response in the host, along with fibrosis. In the chronic phase, the heart is usually the most affected organ, where the amastigotes multiply and form pseudocysts within cardiomyocytes, causing an inflammatory response resulting in cardiac damage. But many other tissues may also be implied, such as lymph nodes, skeletal muscle, liver, spleen, kidney, brain, and digestive tract. The figure was partly designed using Servier Medical Art, provided by Servier, licensed under a Creative Commons Attribution 4.0 unported license (https://creativecommons.org/licenses/by/4.0/).

Infection by *T. cruzi* can cause a wide range of clinical signs that share several similarities among the different species affected. In general, the clinical presentation of CD has two phases—acute and chronic [29,72,73] (Figure 2) (p. 6). Nevertheless, in any stage of CD, clinical signs may vary from absent to severe and life-threatening.

During the acute phase of infection, *T. cruzi* trypomastigotes circulate within the bloodstream and can enter macrophages, disseminating throughout most body tissues The invasion of host cells by trypomastigotes, followed by their transformation into intracellular amastigotes induces a systemic inflammatory response, along with fibrosis. While inflammation is more pronounced in the acute stage, fibrosis predominates during the chronic phase [29,73,74,75,76,77]. The heart is usually the most affected organ [29,75,77,78,79,80,81], but many other tissues may also be implied, such as lymph nodes, skeletal muscle, liver, spleen, kidney, brain, and digestive tract [29,76]. The amastigotes multiply and form pseudocysts within cardiomyocytes, causing an inflammatory response resulting in cardiac damage [77,79,82]. Besides the parasite-induced lesions the innate and immune responses contribute to sustained inflammation, fibrosis, and oxidative stress lesions, causing myocyte necrosis, autonomic dysfunction, microvascular dysfunction, cardiac hypertrophy, and fibrosis, which may culminate in heart failure [13,29,75,76,77].

The CD acute phase is observed in around 1–2% of infected people and occurs within the first few weeks after exposure. It is characterized by a high parasitic load in the bloodstream and lasts a few weeks or months [73,76]. Human patients usually have absent to mild symptoms, such as lymphadenopathy or self-limiting fever [6,78]. Other clinical signs may include body aches (e.g., headache, muscle, abdominal, or chest pain), weakness/fatigue, loss of appetite, diarrhea, vomiting, rash, pallor, respiratory difficulties, swelling of the face or limbs, hepatomegaly, splenomegaly, and tachycardia [6,73,78]. The so-called “chagoma” is a typical sign of CD and is a swelling area near the site of infection. When it is in the eyelid(s) is called “Romaña’s sign” [1,72]. Despite being characteristic, these clinical signs appear in less than 50% of *T. cruzi*-infected people [72,73].

In dogs, clinical signs of acute disease vary largely with the age of diagnosis. In adult dogs diagnosed with CD, survival time is higher and prognosis is more favorable than when the diagnosis occurs in puppies [29,67,83,84]. Puppies are more likely to develop severe signs, such as lethargy, generalized lymphadenopathy, slow capillary refill time with pale mucous membranes, and signs of acute myocarditis with heart failure as well as ascites, weak pulse, hepatomegaly, splenomegaly, and sudden death, while adults mostly present mild signs such as slight depression or low-rising parasitemia [22,67,79,80,82,83,84,85,86,87].

These conclusions align with the long-dated concept of dogs as sentinels for Chagas disease, since they develop similar clinical signs to those found in humans and for many years now have been considered models for scientific purposes [40,87,88].

In rare occasions (<5–10%), acute CD may progress to severe cardiomyopathy [75,89,90,91,92] or meningoencephalitis [93,94,95], generally leading to death [62,78]. These alterations are rare and affect mostly young children or immunocompromised people, such as those taking immunosuppressant therapies or coinfected with HIV [73,95]. The other 90% of infected people resolve acute disease manifestations spontaneously, from which 60–70% remain asymptomatic (the so-called “indeterminate form” [IF]), while 30–40% convert into the symptomatic chronic form, approximately 10–30 years after the initial infection [94]. In dogs, these proportions are not so well established, given their high variability with age and shorter lifetime. However, Nogueira-Paiva and collaborators (2014) [96] and Barr et al. (2009) [29] considered that infected dogs that are more than 6 months old show no signs of acute disease and enter the chronic phase about 30 days after infection. Similar to humans, infected dogs surviving the acute phase will also enter the chronic phase, either asymptomatic (IF) or symptomatic, and clinical signs share several similarities and vary from absent to severe [29,81,97,98].

The chronic phase can last up to the entire life of the host [62,73]. During this phase, parasitemia diminishes since parasites are mainly hidden in the heart and digestive muscles [75,99]. The IF is considered a benign clinical condition, characterized by positive serological and/or parasitological results for *T. cruzi* infection, but absence of clinical manifestations of disease. In humans, there is a consensus that IF patients must have normal electrocardiogram (ECG) readings and normal radiological findings in the heart, esophagus, and colon [13,99,100]. In dogs, IF is not so extensively characterized and is generally attributed to dogs that are seropositive for *T. cruzi* infection and clinically asymptomatic [13,29,81]. In humans, the IF affects approximately 70% of chronic patients, although around 6.9% of IF patients may convert into symptomatic [62,73,101]. The prognosis of human IF is similar to that of healthy individuals with normal ECG readings [101,102].

The symptomatic form of chronic CD may be manifested by cardiac, digestive neurological, and mixed forms [62,73,96,103], depending on the main clinical manifestations shown. Cardiomyopathy is the most frequent and serious (high morbidity and mortality rates) clinical presentation of CD in humans [6,76], being the main cause of human infectious myocarditis worldwide, leading to a substantial public health burden [76,78,104]. Cardiac disease is also the most severe and life threating clinical presentation of CD [22,29,81]. The clinical signs derived from chronic myocarditis include altered heart rate, arrhythmias, cardiomegaly, congestive heart failure, thromboembolism, and cardiac arrest with sudden death. The ECG abnormalities are frequently seen and associated with *T. cruzi* infection, demonstrating cardiac conduction abnormalities such as ventricular arrythmias and atrioventricular block in humans [76,99,104] and dogs [86,105,106,107,108,109]. In dogs, cardiac disease has been the most extensively studied clinical presentation of CD [22,29,81]. In a study with 537 confirmed cases of canine CD in Texas, Kjos et al. (2008) reported “enlarged heart” as the most common clinical observation (33.6%) in dogs diagnosed with CD, followed by lethargy (28.7%), anorexia (23.0%) ascites (22.1%), cardiac conduction disturbances (21.3%), among other abnormalities with lower proportions each [67]. However, there is no compelling research defining the cardiac form as the most prevalent in canine CD, compared with digestive, neurological or other clinical signs. Cardiac alterations generally begin in the right side of the heart, then may progress to the left [29,82,83,110]. Another marker of cardiac disease which has been increasingly studied is troponin I. Studies conducted in dogs [81,82,84] and humans [111,112] with CD have associated increased levels of serum troponin I with heart disease, suggesting that it could be used as biomarker and predictor of disease outcome in such patients. In dogs with CD, increased troponin I has been associated with the presence of heart disease [81,82,84]. In dogs, sensibility of ECG as a diagnostic tool varies among studies. On the one hand, some studies report that ECG abnormalities may be detected in over 76% of seropositive patients [22,80,83]. On the other hand, a case report by Vitt et al. (2016) suggested that sensibility of ECG and also serology may be reduced in acute CD, compared with other markers such as troponin I [82]; furthermore, Kjos et al. (2008) reported only 22.1% of cardiac conduction disturbances detected among 537 dogs confirmed for *T. cruzi* infection by serology (*n* = 444), histopathology [86] and the combination of both methods (*n* = 7) [67]. These differences may also be reflecting a different host immune response. Oral transmission is more likely in dogs due to ingesting infected bugs [29,69,86] and thus being exposed to a higher density of parasites [113]. Carvalho et al. (2019) [107] estimated that 28% of 78 dogs experimentally infected with *T. cruzi* were affected by chronic cardiac signs, 6–9 months post-infection, a percentage that is similar to that reported in humans.

In humans, the digestive, neurological, and mixed forms affect approximately 10% of individuals with chronic CD [81]. The digestive form generally manifests as peristalsis dysfunction and progressive enlargement of the esophagus (megaesophagus), colon (megacolon), or other parts of the intestine [76,78,114], affecting normal digestive function. In humans, difficulties in eating (dysphagia) or defecating (constipation) have been described in CD patients [76,99]. These signs have been associated with chronic inflammation and lesion/degeneration of parasympathetic enteric neurons [76,78,96]. In dogs, there is scarce information concerning digestive signs of CD, which may include decreased appetite and diarrhea [80,82]. Nogueira-Paiva and colleagues (2014) [96] noticed that two different strains of *T. cruzi* that were inoculated in dogs were detected in the esophagus and colon, along with lesions of inflammation and myenteric denervation 30 days post-infection. However, lesions caused by the Be-78 strain persisted until 720 days post-infection and specific lesions of megaesophagus or megacolon were not reported.

Neurological signs in chronic CD derive from the damage of the nervous system, including inflammation, infiltration, and demyelination lesions in the nerves, which affect sensory and motor capabilities [46,76,96], and digestive signs are one of the clinical manifestations of this phenomenon. Sporadically, the central nervous system may be affected, causing dementia, confusion, chronic encephalopathy, and sensory/motor deficits [46,76]. In dogs, neurologic signs are also rare, being associated with multifocal encephalitis associated with parasitic invasion of the neurologic system (pseudocysts). Clinical manifestations include weakness, pelvic limb ataxia, and hyperreflexive spinal reflexes that resemble distemper [29,67,82,115]. Laboratory abnormalities may include anemia, lymphocytosis, hyperproteinemia with hypoalbuminemia and hyperglobulinemia, hypoglycemia, and increased levels of lactate dehydrogenase (LDH), aspartate transferase (AST), ALT, BUN, creatine kinase (CK), creatine kinase myocardial band (CK-MB) and serum troponin I [82,85,88,113,116,117]. Table 2 presents a summary of the similarities and differences between human and canine CD detailed in this section.

## 5. Diagnosis: A Key Step in Tackling Chagas Disease

The large spectrum of different and non-specific clinical signs, considering that the vast majority of infections are asymptomatic, hampers the diagnosis of infection by *T. cruzi* as their etiological cause [1,72,73]. Diagnosis of CD is complex and should integrate information about the epidemiological context, clinical history, clinical signs and/or laboratory abnormalities compatible with the disease and further confirmation of parasite infection through one or more complementary diagnostic methods [13,78,117].

Tests to detect *T. cruzi* infection include direct techniques, which confirm the presence of the parasite or its components, such as parasitological and molecular assays, and indirect techniques that assess the host’s immune response to the parasite by detecting specific humoral immune response [11,73].

Direct quantification of CD incidence in dogs is rare, most likely due to the challenges of collecting longitudinal data. However, a recent study conducted in Texas evaluated *T. cruzi* serology and DNA of 64 dogs at three time points over a year, recording an incidence rate of 30.7 new infections per 100 dogs per year [11]. In contrast, a previous serological survey carried out in the same state by Garcia and colleagues (2016) [118] estimated an overall serological incidence of 3.8% (8 out of 209 samples) in dogs in addition to high infection rates (51–82%) in triatomine vectors. Despite these studies, the epidemiology and seroprevalence of *T. cruzi* infection in companion animals is largely unknown, and canine CD is likely to be underdetected and underreported.

The serological diagnosis allows the detection of parasite antibodies and is considered the primary choice (gold standard) for CD diagnosis in both humans [117,119] and dogs [29,81,82,86] (Table 3). Serological methods include qualitative immunochromatographic tests (ICT), and quantitative tests such as enzyme-linked immunosorbent assay (ELISA), chemiluminescent microparticle immunoassay (CMIA), indirect immunofluorescence (IIF), or hemagglutination inhibition assay (HAI). In dogs and humans, the sensitivity and specificity of serological assays are greater than 90% and increase over infection time [13,76]. Positive serology indicates previous exposure to the parasite, but also a probable current infection as a parasitological cure is considered highly unlikely [29,84,119]. However, sensitivity varies within different serological tests and *T. cruzi* strains being screened and is lower in acute infection [13,82,117,119]. Moreover, the specificity of serological tests can be affected by cross-reactivity with other trypanosomatids, such as *Leishmania* spp., in both humans [120,121] and dogs [113,122,123]. The WHO (2012) [76] stressed the need to develop less invasive and more accurate diagnostic techniques, especially point-of-care tools to improve screening and allow rapid, cost-effective action and treatment. Rapid diagnostic tests have been tested in both humans [124] and dogs [84,125,126], but are currently available for humans only. Nevertheless, they have been used off-label in animal studies and some have shown good accuracy [81,125,126,127]. Additionally, advances in DNA recombination technology have allowed the use of recombinant proteins in immunoassays. IBMP antigens are chimeric recombinant *T. cruzi* antigens, which have shown good diagnostic performance in both humans [128] and dogs [127], including negligible cross-reaction with *Leishmania* spp. [13,128]. In veterinary medicine, the use of serological tests for *T. cruzi* infection is still limited by the species-specific test kits available for most animal species, with few laboratories offering options for dogs [74].

Parasitological diagnosis consists of the direct microscopic observation of *T. cruzi* parasites through cytology, histology, immunohistochemistry, xenodiagnoses, and culture of biological samples, such as blood, host tissues, or even triatomine feces [78,119,129,130]. Parasitology enables confirmation of infection, with a specificity of about 100%, and can be helpful when antibody levels are low, such as in recently infected cases [13,74,76]. In contrast, sensitivity is generally low and decreases in CD’s chronic phase [76,82,87]. Concentration techniques may be used to increase sensitivity, such as the microhaematocrit concentration method (MH), the Strout concentration method as well as the examination of the buffy coat or the pellet (red and white blood cells) obtained after plasma centrifugation [76]. Culture/hemoculture and xenodiagnosis allow amplification of the parasite, but sensitivity remains low (22% in hemoculture and 11% in xenodiagnosis, and are not practical for clinical settings, mostly being performed in research scenarios [131].

**Table 3:** Diagnosis methods for CD. Available tests to identify *T. cruzi* infection include direct techniques, and may be useful to confirm the parasite or its cellular components by parasitological and molecular methods (PCR or qPCR: quantitative polymerase chain reaction of *T. cruzi* nuclear satellite DNA [nDNA] and minicircle kinetoplast DNA [kDNA] and loop-mediated isothermal amplification [LAMP]). Indirect methods can be used based on evaluating the host’s humoral immune response against the parasite (serological methods) such as: (i) enzyme-linked immunosorbent assay (ELISA); (ii) immunochromatographic test (ICT); (iii) chemiluminescent microparticle immunoassay (CMIA); (iv) hemagglutination inhibition assay (HAI) and (v) indirect antibody immunofluorescence reaction test (IFAT).

	Diagnosis	Humans		Dog		Limitation
		Acute	Chronic	Acute	Chronic	
Serological	^+^ ELISA	Yes	Yes	Yes	Yes	Sensitivity varies within different serological tests and *T. cruzi* genetical variability and is lower in acute infection. In acute phase most of humans and dogs are asymptomatic. Cross-reactivity with other trypanosomatids, such as *Leishmania* spp. in endemic overlap area. Serological tests present variability in the different kits. Besides, a high cost of commercially assay available at such as ICT and CMIA.
	^+^ ICT	Yes	Yes	^#^ NA	^#^ NA	
	* CMIA	Yes	Yes	NA	NA	
	^++^ ELISA + HAI ^++^ ELISA + IFAT	Yes	Yes	Yes	^+^ Yes	
Parasitological	Microscopy: Direct observations (stained or fresh blood preparation sample)	Yes		Yes		Parasitemia depends on the phase of the infection. Detection of parasites is primarily applicable during the acute phase of infection. During chronic phase, parasitemia tends to be low and intermittent reducing sensitivity in detection. Parasitemia in domestic dogs vary according to region.
	Artificial xenodiagnosis	Yes	No			
	Haemoculture	Yes		* Yes		
Molecular	PCR, qPCR (nDNA- and kDNA-based qualitative)	Yes	^+^ Yes	^+^ Yes	^+^ Yes	*T. cruzi* DNA detection is solely applicable in acute phase. Parasitemia generally low and intermittent in the chronic phase. Thus, reduce the sensitivity.
	LAMP (*Trypanosoma cruzi* Loopamp kit)	NA	NA			

^+^ Recommended in sero-epidemiological surveys and follow-up. Although, ICT is not recommended in patients screened for Chagas disease (chronic infection) in hemotherapy services (PAHO, 2019). ^++^ Diagnostic gold standard diagnosis, i.e., the combining of two positive serological tests and potentially a third test if the results are conflicting. * Recommended to screen Chagas disease in hemotherapy. NA: Not approved for human and veterinary clinical practice. ^#^ NA: Not approved in clinical veterinary practice. Rapid tests (ICTs) that detect antibodies to *T. cruzi* for the diagnosis of Chagas disease in humans are not currently approved for clinical use in animals. However, few studies were testing Chagas/Bio-Manguinhos Lateral Flow Immunochromatographic Rapid Test (Chagas-LFRT); Trypanosoma Detect™ InBios and CHAGAS STAT-PAK™ in dogs [132].

Molecular methods enable the detection of *T. cruzi* DNA from various biological samples, including triatomine vectors. Similarly, with parasitological methods, it may be important to confirm an active infection and differentiate it from an exposed (seropositive), but non-actively infected animal. Molecular techniques based on PCR are highly specific, but according to the biological sample, sensitivity can be relatively low [13,29,76,131]. Since it can be performed in all tissue samples, it may be more sensitive than histopathology [87], and can provide information on the genetic strain of the parasite, which is useful for research. Eloy and Lucheis [133] have concluded that PCR using TCZ1/TCZ2 primers constitutes a suitable tool for parasite detection in cat and dog hemocultures, and could be used as an enabler for diagnosis. Also, molecular techniques may help to confirm infection in seronegative (recently infected) patients [78].

The development of new molecular-based diagnosis for use in resource-limited areas, such as the isothermal amplification of nucleic acids (LAMP) has the potential to be applied in the detection of *T. cruzi* and *Leishmania* sp. infections. Besuschio and colleagues [134] evaluated LAMP’s capacity to accurately diagnose acute human CD in different epidemiological and clinical scenarios. Their conclusion suggested that the *T. cruzi* Loopamp kit shows promise for the swift detection of *T. cruzi* infection in cases of congenital transmission, acute infection, and Chagas disease reactivation associated with HIV infection. According to the most recent guidelines for CD diagnosis [119], the diagnostic tests recommended varied according to the clinical context/scenario. The guidelines evaluated four main diagnostic methods: ELISA, ICT, CMIA and the diagnostic gold standard method, which is the combination of two positive serological tests (ELISA, HAI, or IIF), and a third test could be added in case of contradictory results. In summary, the guidelines recommend the use of the diagnostic gold standard (rather than ELISA, ICT, or CMIA as single isolated tests) to obtain a definitive diagnosis in patients with suspected chronic *T. cruzi* infection. However, to screen CD in populations, the recommendation is to perform the ELISA or ICT tests as single tests since these assays are easier to implement. However, when the aim is to screen CD in hemotherapy services, the guidelines advise the use of ELISA (highly sensitive kits) or CMIA.

Complementary exams such as ECG and echocardiographic examination are important and have been recommended to screen for cardiac disease in *T. cruzi*-infected patients [22,29,78,87,106,107,108], and serum cardiac troponin I (cTnI) has been increasingly studied as an early biomarker of parasitic myocarditis in dogs with CD [81,82,113]. In dogs, echocardiographic and ECG evaluations are not as sensitive, lacking significant changes in dogs with acute CD [82]. Although not confirming the presence of the parasite, ECG, echocardiography and cTnI may provide helpful information for a more robust diagnosis and characterization of CD [81,82,86,87,106,107,113], and researchers have recommended its use in periodic screening of patients serological positive for *T. cruzi* antibodies, either symptomatic or not [81,82]. Some dog breeds seem to have a genetic predisposition to heart diseases, as is the case with German Shepards, Bulldogs, or Boxers, and in these breeds, the identification of the etiological agent causing cardiac abnormalities should be carefully investigated [29,84].

Therefore, the diagnosis of human and canine CD faces several challenges. For human CD, most diagnostic tests require invasive sampling and accuracy is highly variable according to the clinical phase of infection [70]. In veterinary medicine, the situation worsens since there are few diagnostic options available and standardized for animals, and tests are often discordant [13,44,125]. Additionally, clinical information concerning animals is often less solid or inaccessible, namely in field or wildlife studies [74,78]. However, according to the London Declaration on Neglected Tropical Diseases initiative, any interventions to reduce infection in dogs and improve their overall health may contribute to decreasing the risk of locally acquired human disease [74,135]. Therefore, veterinary CD diagnostics should not be overlooked.

## 6. Tackling CD Control: Current Treatment and Prophylaxis Strategies

Treatment of *T. cruzi* infection is unsatisfactory and challenging. Only two therapeutic options with proven efficacy are available: Benznidazole (BNZ) and Nifurtimox (NFX). Widely used in Latin America, BNZ is a nitroimidazole derivative that induces oxidative stress generating nitrate and oxygen-reactive species, which can cause damage to parasite cellular machinery. Similarly, the nitrofuran NFX generates oxidative metabolites, such as oxygen peroxide free radicals, capable of inactivating the parasite [136,137]. The knowledge of the mechanism of action of a drug is essential for optimizing its therapeutic benefits, minimizing adverse effects, and advancing drug development towards more effective and eventually even *T. cruzi* DTU-specific treatments. The analysis of DTUs has gained traction in recent years, with several published studies examining the DTU impact on patient outcomes [138,139,140]. However, it is important to note that the DTU system alone cannot reliably predict disease outcomes or responses to therapy, and so far, there is no singular outcome associated with any specific DTU.

Although their safety and efficacy profiles are far from ideal, both drugs have been the first-line treatment for about 50 years. BNZ and NFX have beneficial effects in the acute phase of CD, achieving resolution in up to 80% of treated patients, especially children under 14 years old [5]. In contrast, treatment effectiveness in chronic patients continues to be highly debated. In a systematic review, trypanocide therapy was considered optional for adults older than 50 years without advanced cardiomyopathy and for patients with gastrointestinal disease but without advanced cardiomyopathy due to an unclear risk–benefit balance [136,141]. In the chronic phase, NFX achieves resolution rates between 7 and 10% [110,142,143] and BNZ ranges between 2 and 40% [5,144,145]. However, the adverse side-effect profiles have led Brazil and other South American countries to discontinue the production and the clinical application of NFX [145]. Standard doses of BNZ also can cause adverse reactions, such as manifestations of hypersensitivity, generalized edema, fever, muscle pain, bone marrow depression, neurological and sleep disorders, weight loss, nausea, and vomiting, among others [146,147]. Consequently, the rate of suspension or abdication of treatment ranges from 15% to 20% of patients [148,149]. Adding to poor drug tolerability in adult populations with chronic disease, there is also a gap in the definition of adequate cure criteria. The issue lies in the fact that the widely accepted criterion for cure is seroconversion by presenting two negative results from two different conventional serology tests. However, antibodies may persist in the host and existing serological techniques continue to yield positive results for years post-treatment. This challenge persists because, as an intracellular parasite, the presence of parasitemia does not serve as a significant indicator of cure.

Treatment of indeterminate chronic infection is insufficient and does not seem to have a significant impact on the clinical course of the disease [150]. Currently, the international clinical guidelines recommend that anti-parasitic treatment should be offered to adults aged 19 to 50 years who are in the chronic indeterminate stage or have mild to moderate cardiomyopathy, children with congenital or acquired acute disease, immunosuppressed hosts with acute or reactivation of chronic disease, and women of childbearing age to prevent congenital transmissions [5,6,136]. In August 2017, the United States Food and Drug Administration (FDA) approved the first BNZ treatment for CD in children aged 2 to 12 years, while in Europe, it is not yet formally approved [151].

As for canine CD, there is no established protocol for the use of BNZ and NFX. There are few studies that evaluated the effects of BNZ in experimentally induced chronic Chagas disease [152,153]. Preclinical results demonstrated the beneficial effect of etiological treatment in reducing tissue damage and transient parasite burden [153]. However, BNZ therapy did not prevent echocardiographic abnormalities associated with cardiomegaly and instead showed an increase in ventricular size similar between infected, treated, and untreated animals. Altogether, treatment with BNZ was unable to prevent the development and progression of chagasic cardiomyopathy. Therefore, the investment in early diagnosis for human and canine CD is essential to ensure the best therapeutic outcome.

Alternately, specialists in CD chemotherapy recommended the evaluation of drug combinations to explore alternative strategies and reproposing of medical treatment options. However, a study combining the two antiparasitic drugs itraconazole and BNZ failed to reduce parasite-induced lesions in dogs. Furthermore, regarding resistant *T. cruzi* strains, the BNZ–intraconazole formulation was not effective in inducing parasitological cure or sustained reduction in the parasite load in the blood and infected organs during the acute CD phase [154]. Madigan and colleagues (2019) [155] evaluated the clinical, serologic, parasitological, and histologic outcomes of naturally infected dogs treated with a combination of amiodarone and itraconazole for 12 months. The results have demonstrated the improvement of clinical signs, increased survival time, and negative PCR for *T. cruzi* DNA. Despite these results, a recent study reported the sudden death of two dogs with symptomatic chagasic cardiomyopathy after receiving amiodarone and itraconazole in addition to cardiac therapy [156]. The development of new therapeutic options has also been addressed by the scientific community. Some pharmacological classes are especially promising to treat CD such as nitroheterocyclic compounds, inhibitors of sterol biosynthesis, cruzipain inhibitors, aromatic amides, trypanothione reductase inhibitors, ruthenium complexes carrying trypanocidal molecules, oxaboroles and nucleoside derivatives, as detailed in a review by Mazzeti and colleagues [157]. Alternative drugs, such as posaconazole, have demonstrated superior results in the treatment of acute disease compared with BNZ; however, only BNZ had efficacy in the chronic mouse CD model [158]. In a double-blind, randomized, placebo-controlled, dose-finding, proof-of-concept study conducted in Bolivia, the nitroimidazole fexinidazole demonstrated high efficacy in chronic *T. cruzi* infection, even at the lowest tested dose, and at less than 3 days of treatment [159]. Making this a very promising drug alternative for further studies. Likewise, the generation of new regimens of BNZ application in combination with fosravuconazole has been demonstrated to be promising in preclinical studies [160]. However, fosravuconazole monotherapy resulted in only a transient response and no sustained effect in a phase 2 study [161]. The effectiveness of a drug’s trypanocidal activity can be dependent on internal factors of the host (such as genetics, immunocompetence, metabolism, and other chronic conditions) associated with parasite genetic heterogeneity. Consequently, further studies should use a target-based or phenotype-based approach to improve the efficacy of compounds for the treatment of human and canine CD. Undoubtedly, there is an urgent need for effective therapeutic options directed at chronic CD. However, for the time being, and due to the few therapeutic options, preventive measures related to vector ecology for transmission control such as insecticide-treated bed nets or netting and the use of insecticide-treated dog collars still play a crucial role in controlling CD.

## 7. Vaccines for CD: Challenges and Opportunities

Recent advances in the search for control and cure of Chagas disease have been focused on the development of prophylactic and therapeutic vaccines that can integrate an effective strategy for prevention and control of *T. cruzi* transmission by modulating the host’s immune effector mechanisms, culminating in parasite clearance, infection control, and pathogenesis prevention, long-term protection. The accumulated knowledge of the biology and genetics of *T. cruzi*, together with an increased understanding of the host immune response, has led to the development of several vaccine candidates against this parasite.

Several vaccine candidates are being developed using different strategies and tested in animal models, such as live attenuated vaccines, recombinant proteins vaccines, replicating recombinant vector vaccines, DNA, and mRNA vaccines [162,163,164,165,166]. Therapeutic DNA vaccines with plasmid DNA encoding *T. cruzi* antigens of the parasite surface trans-sialidase family (TSA-1, Tc52, or Tc24), which seems to be crucial for parasite evade host immune response have been tested in acute and chronically infected mice with different outcomes. Tc24, TSA-1, and Tc52 reduced parasitemia, controlled myocarditis, and decreased mortality [167,168,169] associated with a rapid expansion of CD4^+^ and CD8^+^ T cell populations [170]. Other studies have addressed the immune response generated by Tc24 and TSA-1 [168,171,172,173,174,175]. Both recombinant proteins induced a strong humoral and cellular immune response in preclinical trials, but these same candidates did not halt cardiomyopathy in infected mice or dogs. Barry et al. (2016) [176] explored the potential for a therapeutic nanoparticle vaccine by encapsulating Tc24 protein in poly (lactic-co-glycolic acid) nanoparticles. Mice infected with a highly lethal H1 strain of *T. cruzi* and then immunized with Tc24-nanoparticles exhibited antigenic specific proliferative cytotoxic (CD8^+^) T cells and Th1 immune response associated with increased production of antigen-specific interferon (IFN)-γ by splenocytes and high IgG2a titers. There was also parasitemia reduction, low inflammatory cell infiltrate, and a decrease in the parasite burden of cardiac tissue. In another study, the same group used the recombinant Tc24 protein adjuvanted by the Toll-like receptor 4 agonist E6020 to immunize mice chronically infected with the H1 strain of *T. cruzi* and showed that 60% of therapeutically vaccinated mice had untraceable parasites accompanied by a decrease in cardiac fibrosis [177].

Due to CD complexity, the use of bioinformatic resources to screen parasite genomes aiming to identify potential vaccine candidates has been carried out by several researchers. Potential candidate antigens selected by screening the *T. cruzi* genome sequence database [178,179] were then analyzed in vitro considering biological parameters. Of the in silico selected antigens, three intracellular candidates (TcG1, TcG2, and TcG4) phylogenetically conserved within the parasite, recognized by host IgG antibody and able to induce proinflammatory CD8^+^ T cell response were used for vaccine development [178,179,180]. It was further confirmed that these antigens were recognized by antibodies and CD8^+^ T cells of a variety of *T. cruzi*-infected hosts [181]. Furthermore, when administered individually as a DNA prime/boost vaccine in mice, these antigens induced trypanolytic activity, a characteristic that has been associated with a protective immune response against *T. cruzi* [178].

The protective efficacy of a *T. rangeli* booster vaccine with primed/inactivated DNA (TcVac4) against *T. cruzi* infection and Chagas disease in a canine model has also been addressed. The use of heterologous DNA priming vaccine/inactivated microorganism booster [181] or DNA booster vaccine/inactivated microorganism priming [174] has been previously reported with promising results. In this scientific approach, inactivated *T. rangeli* was used as a booster dose of the vaccine for several reasons: (i) *T. cruzi* lysates were first tested and demonstrated to provide limited or no protection; (ii) it was considered that *T. rangeli* exhibits significant homology (>60%) with the *T. cruzi* proteome [182,183] but is not pathogenic for mammals [184,185] and (iii) mice immunized with *T. rangeli* fixed in glutaraldehyde elicited B and T responses that recognized *T. cruzi* antigens [186,187]. Mice immunized with *T. rangeli* were able to control *T. cruzi* challenge, showing a significant reduction in parasitemia, the absence of histopathological lesions, and low mortality [186,187]. The *T. rangeli*-based vaccine has also been tested in dogs with positive results. Dogs immunized with glutaraldehyde-inactivated *T. rangeli* epimastigotes exhibited reduced parasitemia following *T. cruzi* challenge and were less infective to triatomines when compared to unvaccinated controls [188].

Co-delivery of parasite antigens as a DNA vaccine induced additive immunity and a greater degree of protection against *T. cruzi* infection in mice [179]. When tested in dogs, TcVac1, which is constituted by antigen-encoding plasmids (pCDNA3.TcG1, pCDNA3.TcG2, and pCDNA3.TcG4) and IL-12 and GMCSF expression plasmids, induced a parasite-specific IgM and IgG response, but phagocyte activity was suppressed, resulting in parasite escape and dissemination into tissues that lead to cardiac histopathological abnormalities, remained infective to triatomines [180]. A further similar approach with TcVac4 (DNA-prime/*T. rangeli*-boost) vaccine in dogs provided control of cardiac pathology, resistance to disease progression, and decreased parasite transmission to triatomines [189]. However, it was not possible in any of the studies carried out to achieve sterile immunity against *T. cruzi* by vaccination.

Recently, immunization of mice with heterologous mRNA Tc24 protein [190]. This new approach to RNA vaccines is based on a new generation of RNA-based vaccines that have demonstrated the ability to induce protective immunity, inducing strong antigen-specific CD8^+^ T cell responses and effective responses of CD4^+^ T cells [191,192]. In this study, it was possible to verify that heterologous mRNA protein vaccination with Tc24 mRNA to prime and Tc24-C4 protein (a genetically engineered polypeptide construct free of cysteines) to boost promotes a cellular immune response against *T. cruzi*, mainly characterized by an increased level of polyfunctional CD8^+^ T cells.

Although the results of these vaccine candidates are encouraging, to date, no anti-*T. cruzi* vaccine has achieved the expected results of producing sterile immunity in dogs or mice. Despite the different strategies that are currently being pursued by researchers, the challenges of developing a therapeutic and/or prophylactic vaccine for human or canine CD are immense. In the vast majority of the studies conducted, significant control of the infection is achieved, with induction of protective immunity and parasitemia reduction. However, blocking the development of cardiac fibrosis and cardiomyopathies and complete parasite clearance remain major challenges to be addressed.

## 8. Final Considerations and Future Perspectives

*Trypanosoma cruzi* is a genetically and ecologically heterogeneous parasite associated with different geographic regions and zoonotic transmission cycles throughout the Americas, presenting different epidemiologic importance and diverse clinical outcomes [139,193]. Despite recent efforts to control CD, much remains unknown and further studies that take into account the complexity of the disease and the current knowledge of parasite–host interactions are needed to evaluate potential new immunotherapies. Moreover, little is known about the factors influencing the disease progression and the role played by an immune response in parasite reactivation and further research should be conducted. Recently, Gil-Jaramillo and colleagues (2021) [194] carried out a comparative RNA-sequencing-based transcriptome analysis of infected human monocyte-derived dendritic cells and demonstrated a new and unexplored pathway process during the first hours of *T. cruzi*–host interaction, similar to anti-viral immune response. These discoveries highlight the close evolutionary relationship between *T. cruzi* and its host’s immune system in order to successfully invade and survive in the host, completing their life cycle. Further, the immunomodulatory functions played by extracellular vesicles produced by the parasite and their potential to contribute to the development of new prophylactic or therapeutic tools against trypanosomatids have been attracting the attention of the scientific community [195,196,197] and should be taken into consideration as a possible innovative control strategy. Currently, the therapeutic approach is focused only on the control of the parasite load and is not sufficient to prevent the progression of the disease to the chronic phase. As there is no defined treatment for CD in dogs or prevention strategies, an immune-precision therapy against the parasite to prevent severe disease should be the focus of future research. In 2021, Mazzeti et al. [157] reviewed, in detail, some studies using innovative experimental treatment strategies, testing new drug candidates and innovative drug associations, and even designing new drug delivery systems to improve drug stability. Although some studies show promising results, still, the real effectiveness of new therapies in humans or animals is not yet established.

The present review highlights canine Chagas disease from different perspectives. The epidemiological role of the dog in CD has been strengthened in recent years, as dogs are likely to be the predominant animal reservoir of *T. cruzi* for human populations and can act as sentinels, since dogs are highly susceptible to *T. cruzi* infection and can develop high parasitemia, facilitating the parasite transmission to the vector. Generally, dogs present the same infection pattern as humans. However, the experimental infection of dogs with strains from South and Central America has revealed some differences in disease outcomes related to *T. cruzi* strain types, including intensity and timing of peak parasitemia, as well as cardiac pathology, thus revealing a complex and dynamic. A One Health perspective recognizes the critical need to protect wildlife, pets/companion animals and human populations from infectious diseases. By improving overall health, the benefit of reducing the risk of locally acquired CD disease will improve public health. This will undoubtedly have a socio-economic impact on the populations affected by CD. 

## Figures and Tables

**Table 1:** *Trypanosoma cruzi* main host species and respective discrete typing units (DTUs) and geographic localization on the American continent. Virtually all mammals are susceptible to *T. cruzi* parasitic infection, although dogs have been playing a critical role in the transition from the sylvatic to peridomestic parasite life cycle.

Host	Specie	DTUs	Geographic Location	References
**Sylvatic animals**				
Opossum Rat Tamandua Primate Armadillo Coati Wild rodents	*Didelphis albiventris* *Didelphis marsupialis* *Didelphis aurita* *Rattus rattus* *Tamandua tetradactyla* *Saguinus midas* *Dasypus novemcinctus* *Euphractus sexcinctus* *Nasua nasua*	TcI TcI TcI and TcII TcI TcI TcI TcIII TcIII TcIV	Argentina Colombia (Vichada department); Venezuela (Anzoátegui state); Brazil Venezuela (Anátegui state) Brazil Paraguay; Brazil Brazil Brazil	[58,59,60]
**Domestic animals**				
**Dog**	*Canis lupus familiaris*	TcI and TcIV TcII, TcV and TcVI * TcI, TcII, TcIV and TcVI TcI/TcII and TcI/TcIV ** TcVI TcI TcI, TcII TcI TcIII/V *** TcIII/V/VI ***	USA (southern Louisiana) USA (southern Louisiana) Colombia (Boyacá department) Argentina Colombia (Vichada department) Venezuela Venezuela (Anzoátegui state) Colombia (Antioquia department) Brazil Brazil	[46,57,58,59,60,61,62,63]
**Human**	*Homo sapiens*	TcI TcI, TcII, TcIII and TcIV TcV	Venezuela (Anzoátegui state) Brazil Argentina; Chile	[58,60,64]

* First time detection ** Mixed infections *** Possible hybridization or co-infection.

**Table 2:** Clinical signs of acute and chronic CD in humans and dogs.

	Humans	Dogs
**Acute CD**	1–2% of infected people Occurs within first few weeks after exposure, lasts up to few weeks/months.	Clinical signs are generally milder and prognosis better along with age of diagnosis
**Parasitaemia**	High	Low rising (adults)
**Absent to mild clinical signs**	Most common presentation of acute CD. When present, clinical signs include: lymphadenopathy, self-limiting fever. In 90% of acute CD cases, clinical signs resolve spontaneously.	Dogs > 6 months old and adults: slight depression
**Other clinical signs**	<50% of infected people may present: body aches (headache, muscle, abdominal, or chest pain), weakness/fatigue, loss of appetite, diarrhea, vomiting, rash, pallor, respiratory difficulties, swelling of the face or limbs (“Chagoma”, “Romaña’s sign”), hepatomegaly, splenomegaly, tachycardia	
**Severe clinical signs**	In <5–10% of acute CD cases (mostly young children and immunocompromised people), clinical signs progress into: “Acute Chagas cardiomyopathy”—cute myocarditis, heart failure Meningoencephalitis Death	Mostly in puppies: lethargy, generalized lymphadenopathy, slow capillary refill time with pale mucous membranes, acute myocarditis with heart failure (ascites, weak pulse, hepatomegaly, splenomegaly, sudden death)
**Chronic CD:**	can last up to the entire life of the host	Dogs > 6 months old (without acute CD) enter the chronic phase about 30 days post infection
**Asymptomatic chonic CD (IF): absence of clinical signs**	60–70% of chronic CD	
**Symptomatic chronic CD:**	30–40% of chronic CD (10–30 years post infection) + 6.9% of asymptomatic (IF) patients	(absent to severe signs, similar to humans) 28% of experimentally infected dogs, 6–9 months post infection
**Parasitaemia**	Diminished	
**Cardiac signs**	“Chronic Chagas cardiomyopathy/myocarditis”: –The most frequent and serious form of CD in humans –Altered heart rate, arrhythmias, cardiomegaly, congestive heart failure (thromboembolism, cardiac arrest, sudden death). –Increased troponin I	Chronic myocarditis: –ECG abnormalities (>76% of seropositive dogs): ventricular arrythmias, atrioventricular block –Increased troponin I
**Digestive signs**	–10% of chronic CD patients ^1^ Peristalsis dysfunction, megaesophagus, megacolon, dysphagia, constipation	–Decreased appetite, diarrhea –inflammation and myenteric denervation in esophagus and colon 30 days post experimental infection, without signs of megaesophagus or megacolon
**Neurological**	–10% of chronic CD patients ^1^ –Inflammation, infiltration, and demyelination affecting sensory and motor capabilities (including digestive function) –Central nervous system lesions (sporadic) may cause: dementia, confusion, chronic encephalopathy, and sensory/motor deficits.	–Rare –multifocal encephalitis with *T. cruzi* pseudocysts; weakness, pelvic limb ataxia, and hyperreflexive spinal reflexes
**Mixed**	–10% of chronic CD patients ^1^	
**Laboratory abnormalities:**		Anemia, lymphocytosis, hyperproteinemia with hypoalbuminemia and hyperglobulinemia, hypoglycemia, and increased levels of lactate dehydrogenase (LDH), aspartate transferase (AST), ALT, BUN, creatine kinase (CK), creatine kinase myocardial band (CK–MB) and serum troponin.

^1^ Digestive, neurological and mixed forms comprise 10% of chronic CD patients.
